# Effect of blood lipid variability on mortality in patients with type 2 diabetes: a large single-center cohort study

**DOI:** 10.1186/s12933-021-01421-4

**Published:** 2021-11-25

**Authors:** Mu-Cyun Wang, Chia-Ing Li, Chiu-Shong Liu, Chih-Hsueh Lin, Shing-Yu Yang, Tsai-Chung Li, Cheng-Chieh Lin

**Affiliations:** 1 Department of Family Medicine, China Medical University Hospital, No. 2, Yude Rd., North Dist., Taichung, 404332 Taiwan (R.O.C.); 2 School of Medicine, College of Medicine, China Medical University, No.91, Hsueh-Shih Road, North Dist., Taichung, 404333 Taiwan (R.O.C.); 3 Department of Geriatrics and Gerontology, National Taiwan University Hospital Hsin-Chu Branch, No. 25, Ln. 442, Sec. 1, Jingguo Rd., North Dist., Hsinchu City, 300195 Taiwan (R.O.C.); 4 Department of Medical Research, China Medical University Hospital, No. 2, Yude Rd., North Dist., Taichung, 404332 Taiwan (R.O.C.); 5 Department of Public Health, College of Public Health, China Medical University, No. 100, Sec. 1, Jingmao Rd., Beitun Dist., Taichung, 406040 Taiwan (R.O.C.); 6 Department of Healthcare Administration, College of Medical and Health Science, Asia University, No. 500, Lioufeng Rd., Wufeng Dist., Taichung, 413305 Taiwan (R.O.C.)

**Keywords:** Type 2 diabetes, Blood lipid variability, All-cause mortality, Cardiovascular mortality

## Abstract

**Background:**

Dyslipidemia is a major cardiovascular risk factor and common in diabetes patients. Most guidelines focus on optimal lipid levels, while variation of lipid profiles is far less discussed. This study aims to investigate the association of visit-to-visit variability in blood lipids with all-cause, cardiovascular, and non-cardiovascular mortality in patients with type 2 diabetes.

**Methods:**

We identified 10,583 type 2 diabetes patients aged ≥ 30 years with follow-up ≥ 3 years and who participated in the Diabetes Care Management Program at a medical center in Taiwan. Variability in lipid profiles within 3 years after entry was calculated using coefficient of variation. Cox proportional hazard models were used to evaluate lipid variability in relation to subsequent mortality.

**Results:**

Over a mean follow-up of 6.4 years, 1838 all-cause deaths (809 cardiovascular deaths) were observed. For each 10% increase in variability in high-density lipoprotein cholesterol, low-density lipoprotein cholesterol, and total cholesterol, the hazard ratios (95% confidence intervals) of all-cause mortality were 1.30 (1.22–1.37), 1.05 (1.01–1.09), and 1.10 (1.03–1.16), respectively; those of cardiovascular mortality were 1.27 (1.16–1.39), 1.08 (1.02–1.15), and 1.16 (1.07–1.27), respectively. Each 10% increase in high-density lipoprotein cholesterol variability conveyed 31% greater risk of non-cardiovascular mortality. High variability in total cholesterol and low-density lipoprotein cholesterol increased all-cause mortality in subgroups of nonsmoking, regular exercising, non-dyslipidemia, and more severe status of diabetes at baseline.

**Conclusions:**

Blood lipid variability except for triglyceride variability was associated with all-cause and cardiovascular mortality in patients with type 2 diabetes.

**Supplementary Information:**

The online version contains supplementary material available at 10.1186/s12933-021-01421-4.

## Background

The prevalence of diagnosed diabetes has increased substantially over the past 30 years, and this situation caused a great burden in healthcare systems [1–3]. Diabetes increases the risk of cardiovascular disease (CVD) by two folds on average, and earlier onset of diabetes is associated with higher mortality rate, which is mostly attributed to CVD mortality [4, 5]. Dyslipidemia is a common comorbidity in diabetes patients, and it affects 60–70% of total diabetes patients and up to 90% of type 2 diabetes patients [6]. Diabetes mellitus (DM) and dyslipidemia are recognized as classic risk factors for CVD [7, 8]. Blood lipids play an essential role in atherosclerosis and the progression of CVD, and they may mediate the association between diabetes and CVD [9]. Abnormal lipid profiles have also increased CVD and all-cause mortality [10, 11].

Recently, intraindividual variability of risk factors over time as a contributor of adverse cardiovascular outcomes and mortality has attracted attention [12]. A meta-analysis of cohort studies showed that visit-to-visit variability in systolic blood pressure was associated with CVD and all-cause mortality [13]. Systolic blood pressure variability was also found to be associated with all-cause mortality independent of average blood pressure in patients with type 2 diabetes [14]. A meta-analysis showed blood glucose variability was associated with mortality in critically ill patients [15]. Further studies showed higher variability in blood glucose increased the risks of Alzheimer disease [16], deteriorating left cardiac structure and systolic function [17], and all-cause mortality in patients with type 2 diabetes [18].

Variability in total cholesterol (TC) was first found to be associated with CVD and mortality in the Framingham Heart Study [19]. Two Korean studies demonstrated that TC variability was associated with nonfatal CVD and all-cause mortality in nationwide population [20] and hospital-based patients undergoing percutaneous coronary intervention [21]. The latter also revealed similar patterns for other lipid components, including low-density lipoprotein cholesterol (LDL-C), high-density lipoprotein cholesterol (HDL-C), and non-HDL-C [21]. In addition, a Chinese cohort study found higher variability in TC increased the risk of all-cause and CVD mortality, but not cancer mortality [22]. However, fewer studies focused on diabetes patients. A Hong Kong study showed the association of variability in LDL-C, TC to HDL-C ratio, and triglycerides (TG) with incident CVD and mortality in type 2 diabetes patients without pre-existing CVD [23]. A study in Taiwan showed standard deviation (SD) of LDL-C, but not that of HDL-C or TG, was independently associated with CVD risk in patients with type 2 DM [24]. These restricted data suggest not only absolute value, but also variability is essential in lipid control for diabetes patients. We still need additional consistent data supporting the effect of lipid variability on CVD and non-CVD mortality separately before conducting an intervention study to confirm benefit from targeting for lipid variability.

This study aims to investigate the visit-to-visit variability in TC, HDL-C, LDL-C, and TG in relation to all-cause, CVD, and non-CVD mortality in patients with type 2 DM.

## Methods

### Study population

A retrospective cohort study was conducted among the participants of the Diabetes Care Management Program (DCMP) at China Medical University Hospital (CMUH), a medical center in Taichung, Taiwan. The DCMP is a case-management program initiated by the Bureau of National Health Insurance since 2002. The DCMP provided financial incentive for clinicians to facilitate more comprehensive care for diabetes patients, including annual health education and assessment by care managers and nutritional therapy practitioners, annual eye examination, and four annual laboratory tests. All patients with a clinical diagnosis of DM, based on the American Diabetes Association criteria (International Classification of Disease, 9th Revision, Clinical Modification [ICD-9-CM] code 250), were invited to participate. Upon entry of the DCMP, the participants underwent a series of laboratory tests and body measurements, and they were required to complete a questionnaire administered by a case-management nurse to ascertain disease status and complication history.

A total of 18,373 adults were enrolled in the DCMP at CMUH in the period of 2002–2015. After exclusion of 448 patients who had type 1 diabetes or gestational diabetes and 504 patients who were younger than 30 years old, 17,421 eligible participants were included. We further excluded 6,385 subjects who had enrolled in DCMP for less than 3 years and thus could not provide 3-year visit-to-visit variation in lipids. A total of 453 subjects with incomplete data of baseline demographics, diabetes-related factors, comorbidities, and laboratory tests were also excluded. In the end, 10,583 participants remained for analysis. The flowchart of study recruitment is shown in Additional file 1: Fig S1. This study was approved by the Human Research Committee of China Medical University Hospital (CMUH108-REC2-153). Informed consent was not required because the study was a secondary data analysis of de-identified data released for research purposes.

### Data for baseline and follow-up assessments

All participants underwent assessment upon entry, which included a series of blood tests, urine tests, eye tests, foot tests, and body measurements. A case-management nurse administered a standardized and computerized questionnaire to record diseases, medication histories, and lifestyle behaviors. After a 12 h overnight fast, blood was drawn in the morning and sent for analysis within 4 h post collection. All participants were followed up regularly every 3–6 months, and they underwent the same tests per year of follow-up, as they did at baseline.

Sociodemographic factors, lifestyle behaviors, and anthropometric measurements were retrieved from the DCMP database. The information included age, sex, smoking status, alcohol consumption status, exercise habits, body mass index (BMI), duration of diabetes, and types of diabetes treatment. Diabetes duration was calculated as the number of days between the date of diabetes onset and entry of DCMP, divided by 365 days. HDL-C, LDL-C, TG, TC, fasting plasma glucose (FPG) and hemoglobin A1c (HbA1c) at the entry of DCMP were retrieved. HDL-C, LDL-C, TG, TC and FPG were analyzed by a biochemical auto-analyzer (Beckman Coulter Synchron system, Lx-20, Fullerton, CA, USA) at the Clinical Laboratory Department of CMUH. HDL-C and LDL-C levels were measured via a direct method, while TC and TG levels via an enzymatic colorimetric method. FPG was measured using a sodium fluoride tube, which contained 5 mg of sodium fluoride to inhibit glucose metabolism and 4 mg of potassium oxalate to chelate calcium and prevent coagulation. HbA1c was analyzed using a boronate affinity high-performance liquid chromatography assay. We further recorded comorbidities including hypertension, dyslipidemia, stroke, and coronary artery disease. DM complications like severe hypoglycemia, peripheral neuropathy, nephropathy, diabetic ketoacidosis, and hyperglycemic hyperosmolar nonketotic coma were also included as confounders. Variability in blood lipid measurements within 3 years after entry were calculated for HDL-C, LDL-C, TG, and TC using coefficient of variation (CV). The CV is the ratio of the SD to the mean, expressed as a percentage by multiplying by 100. Based on this definition, at least two readings of a lipid variable are required to determine the CV.

### Outcome ascertainment

The primary outcomes were all-cause, CVD, and non-CVD deaths. Mortality of participants was determined by a computer linkage with a unique identification number to the death records from the Health and Welfare Data Science Center database. The follow-up time was determined by calculating the days from the date of recruitment to the date of death or to the end of the study.

### Statistical analysis

The baseline characteristics were reported and compared across mortality status by the *t* test for continuous variables, and the chi-squared test for categorical variables. The CV values within the first 3 years for enrollees with at least two measurements of HDL-C, LDL-C, TG, and TC were calculated, and then, they were divided by the square root of the ratio of total visits divided by total visits minus 1 to adjust for the possible effect that the number of visits may have on variation. Cox proportional hazard models were used to examine the blood lipid variability in relation to subsequent mortality from all causes, CVD, and non-CVD. The hazard ratios (HRs) and their 95% confidence intervals (CIs) were presented with multivariate adjustment for age, sex, smoking, alcohol drinking, exercising, BMI, duration of diabetes, types of diabetes treatment, FPG, HbA1c, HDL-C, LDL-C, TG, and TC, in addition to a series of comorbidities and complications. Restricted cubic spines were used to explore nonlinearity between lipid variability and all-cause mortality. To investigate differences in the effect of lipid variability on all-cause mortality for different patient characteristics, enrollees were classified into subgroups by age, sex, smoking status, exercise habit, BMI, duration and treatment of diabetes, indices of diabetes (FPG and HbA1c), and medical history (hypertension and dyslipidemia). For the purpose of sensitivity analysis, lipid profiles were categorized into quartiles and the result was compared with the original one. The two-tailed tests were used, and *p* < 0.05 was considered to indicate statistical significance. All statistical analyses were performed using SAS version 9.4 (SAS, Cary, NC).

## Results

A total of 10,583 diabetes patients were followed up for a mean of 6.4 years. 1,838 all-cause deaths, 809 CVD deaths, and 1,029 non-CVD deaths were observed, which resulted in crude incident density rates of 27.32, 12.03, and 15.30 per 1000 person-years, respectively. The baseline characteristics are shown in Table 1 according to mortality status in patients with type 2 DM. Individuals who died were older, more likely to be females and smokers, and having more exercise and lower BMI. Considering the status of diabetes, those who died had significant longer duration of the disease, increased proportion of treatment, and shift from oral anti-diabetic agent to additive insulin injection. They also had higher risk for comorbidities and complications of diabetes, higher FPG and HbA1c, and lower HDL-C levels.

**Table 1:** The comparisons of baseline sociodemographic factors, life style behaviors, diabetes-related variables, complications and blood biochemical indices according to mortality status in 10,583 patients with type 2 diabetes

Variables	Mortality status		*p* value
	Alive (n = 8745)	Dead (n = 1838)	
Sociodemographic factors			
Gender			0.02
Female	4516 (51.64)	1003 (54.57)	
Male	4229 (48.36)	835 (45.43)	
Age (years)	57.48 ± 11.18	67.29 ± 10.54	< 0.001
Life style behaviors			
Smoking	1456 (16.65)	343 (18.66)	0.04
Alcohol drinking	721 (8.24)	142 (7.73)	0.49
Exercising	4459 (50.99)	1005 (54.68)	0.004
BMI (kg/m^2^)	26.08 ± 4.13	25.26 ± 3.87	< 0.001
Diabetes-related variables			
Duration of diabetes (years)	5.39 ± 6.33	8.91 ± 8.35	< 0.001
Types of diabetes treatment			< 0.001
Oral hypoglycemic drug	7312 (83.61)	1482 (80.63)	
Inject insulin	125 (1.43)	26 (1.41)	
Both	766 (8.76)	260 (14.15)	
No	542 (6.20)	70 (3.81)	
Complications			
Hypertension	2613 (29.88)	837 (45.54)	< 0.001
Dyslipidemia	1686 (19.28)	478 (26.01)	< 0.001
Stroke	278 (3.18)	153 (8.32)	< 0.001
Coronary artery disease	412 (4.71)	198 (10.77)	< 0.001
Severe hypoglycemia	52 (0.59)	38 (2.07)	< 0.001
Peripheral neuropathy	594 (6.79)	311 (16.92)	< 0.001
Nephropathy	382 (4.37)	252 (13.71)	< 0.001
DKA	34 (0.39)	13 (0.71)	0.09
HHNK	60 (0.69)	38 (2.07)	< 0.001
Blood biochemical indices			
Fasting plasma glucose (mg/dL)	151.12 ± 52.4	161.57 ± 66.59	< 0.001
HbA1c (%)	7.80 ± 1.70	8.06 ± 1.86	< 0.001
High-density lipoprotein (mg/dL)	43.67 ± 11.66	41.11 ± 11.66	< 0.001
Low-density lipoprotein (mg/dL)	111.97 ± 34.63	110.87 ± 35.69	0.22
Triglycerides (mg/dL)	165.71 ± 237.63	171.49 ± 212.54	0.30
Total cholesterol (mg/dL)	187.99 ± 43.32	188.48 ± 45.15	0.67

Continuous variables in mean ± SD and categorical variables in number (%)

The Student’s t-test for continuous variables and the chi-squared test for categorical variables to calculate *p* values

**Fig. 1:**
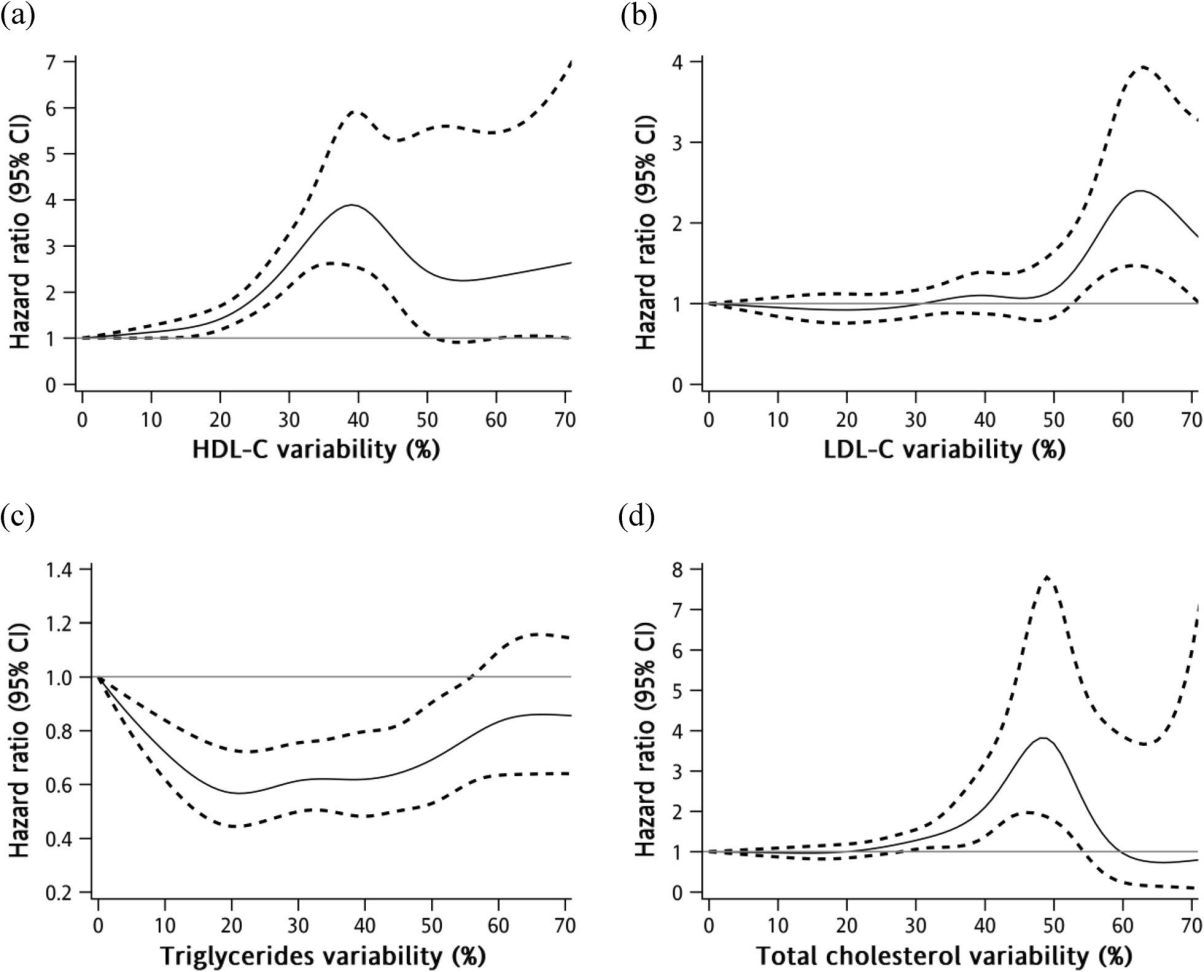
Restricted multivariable cubic spline plots for **a** HDL-C variability, **b** LDL-C variability, **c** triglycerides variability and **d** total cholesterol variability in relation to all-cause mortality

Table 2 shows HDL-C, LDL-C, TG, and TC variability in relation to all-cause, CVD, and non-CVD mortality. In the age and sex-adjusted model, variability in all lipid components significantly increased all-cause mortality and CVD mortality. In both multivariate models, variability in HDL-C, LDL-C, and TC significantly increased all-cause and CVD mortality. In all models, only variability in HDL was associated with non-CVD mortality. In multivariate model with adjusted demographic factors and comorbidities, the HRs (95% CIs) of all-cause mortality were 1.30 (1.22, 1.37), 1.05 (1.01, 1.09), and 1.10 (1.03, 1.16) for each 10% increase in HDL-C, LDL-C, and TC variability, respectively. The HRs (95% CIs) of CVD mortality were 1.27 (1.16, 1.39), 1.08 (1.02, 1.15), and 1.16 (1.07, 1.27) for each 10% increase in HDL-C, LDL-C, and TC variability, respectively. The HR (95% CI) of non-CVD mortality was 1.31 (1.22, 1.42) for each 10% increase in HDL-C variability. In addition, the restrictive cubic spine regression model demonstrated a consistent but nonlinear association of variability in HDL-C and LDL-C, rather than TG, with all-cause mortality, as shown in Fig. 1.

**Table 2:** Hazard ratios of mortality for blood lipid variability in patients with type 2 diabetes

	n	Case	Person-years	IR*	Age and sex adjusted model		Multivariate model 1		Multivariate model 2	
					HR (95% CI)†	*p* value	HR (95% CI)†	*p* value	HR (95% CI)†	*p* value
All-cause mortality										
HDL-C variability	10,583	1838	67,271.91	27.32	1.35 (1.28, 1.43)	< 0.001	1.31 (1.23, 1.38)	< 0.001	1.30 (1.22, 1.37)	< 0.001
LDL-C variability	10,583	1838	67,271.91	27.32	1.07 (1.03, 1.11)	< 0.001	1.04 (1.00, 1.08)	0.04	1.05 (1.01, 1.09)	0.02
TG variability	10,583	1838	67,271.91	27.32	1.05 (1.02, 1.07)	0.001	1.01 (0.98, 1.04)	0.72	1.01 (0.98, 1.04)	0.56
TC variability	10,583	1838	67,271.91	27.32	1.12 (1.06, 1.19)	< 0.001	1.09 (1.03, 1.16)	0.005	1.10 (1.03, 1.16)	0.003
Expanded CVD mortality										
HDL-C variability	10,583	809	67,271.91	12.03	1.33 (1.22, 1.45)	< 0.001	1.28 (1.17, 1.40)	< 0.001	1.27 (1.16, 1.39)	< 0.001
LDL-C variability	10,583	809	67,271.91	12.03	1.11 (1.05, 1.18)	< 0.001	1.08 (1.02, 1.14)	0.01	1.08 (1.02, 1.15)	0.007
TG variability	10,583	809	67,271.91	12.03	1.08 (1.03, 1.12)	< 0.001	1.04 (0.99, 1.08)	0.11	1.04 (1.00, 1.09)	0.06
TC variability	10,583	809	67,271.91	12.03	1.22 (1.13, 1.33)	< 0.001	1.16 (1.07, 1.27)	< 0.001	1.16 (1.07, 1.27)	< 0.001
Non-expanded CVD mortality										
HDL-C variability	10,583	1029	67,271.91	15.30	1.37 (1.27, 1.47)	< 0.001	1.32 (1.23, 1.42)	< 0.001	1.31 (1.22, 1.42)	< 0.001
LDL-C variability	10,583	1029	67,271.91	15.30	1.04 (0.98, 1.09)	0.19	1.01 (0.96, 1.07)	0.62	1.02 (0.97, 1.07)	0.51
TG variability	10,583	1029	67,271.91	15.30	1.02 (0.98, 1.06)	0.31	0.98 (0.94, 1.02)	0.37	0.98 (0.95, 1.03)	0.44
TC variability	10,583	1029	67,271.91	15.30	1.05 (0.97, 1.13)	0.27	1.03 (0.95, 1.12)	0.46	1.04 (0.96, 1.13)	0.31

^*^Number of incident cases per 1,000 person-years

^†^For 10% increase in coefficient of variation

Multivariate model 1 adjusted for age, sex, smoking, alcohol drinking, exercising, body mass index, duration of diabetes, types of diabetes treatment, fasting plasma glucose, hemoglobin A1c, HDL-C, LDL-C, TG and TC

Multivariate model 2 adjusted for hypertension, dyslipidemia, stroke, coronary artery disease, severe hypoglycemia, peripheral neuropathy, nephropathy, diabetic ketoacidosis, and hyperglycemic hyperosmolar nonketotic coma, in addition to the variables in the first multivariate model

**Fig. 2:**
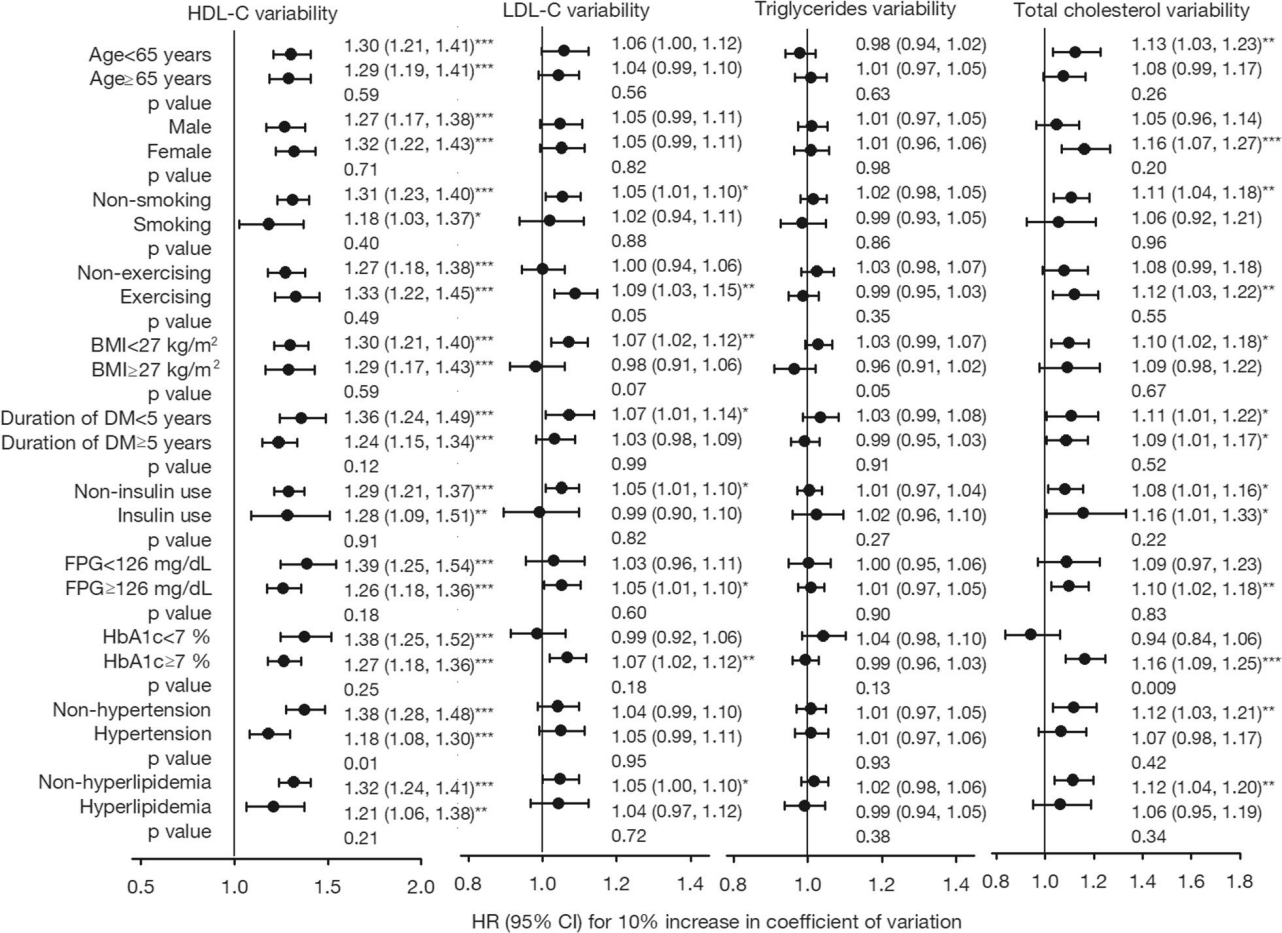
Hazard ratios of all-cause mortality for blood lipid variability in subgroups of patients with type 2 diabetes. Adjusted for age, sex, smoking, alcohol drinking, exercising, BMI, duration of diabetes, types of diabetes treatment, FPG, HbA1c, HDL-C, LDL-C, triglycerides, total cholesterol, hypertension, dyslipidemia, stroke, coronary artery disease, severe hypoglycemia, peripheral neuropathy, nephropathy, diabetic ketoacidosis, and hyperglycemic hyperosmolar nonketotic coma. A separate model fitted for each subgroup. Within subgroups: *, *p* < 0.05; **, *p* < 0.01; ***, *p* < 0.001. The *p* value for interaction at the bottom of each subgrouping variable

The subgroup analyses were reported using forest plots, as shown in Fig. 2. Consistently increased risks of all-cause mortality were observed for higher HDL-C variability in subgroups. In general, the association of LDL-C variability with all-cause mortality attenuated compared with that of TC variability. TC variability was associated with all-cause mortality particularly in enrollees under 65 years of age and females. Higher LDL-C variability and TC variability increased all-cause mortality in enrollees who took exercise regularly and never smoked. LDL-C variability was associated with all-cause mortality for DM patients who was diagnosed within 5 years and had not used insulin yet. Meanwhile, the association between TC variability and all-cause mortality was irrespective of the duration and treatment of DM. LDL-C variability and TC variability were associated with all-cause mortality for DM patients with baseline FPG≧126 mg/dL (7.0 mmol/l) and HbA1c≧7% (53 mmol/mol), but without dyslipidemia. The *p* values for interaction of subgroup analyses were reported in Fig. 2, indicating HbA1c was an effect modifier for the association between TC variability and all-cause mortality. In the same manner, the effect modification of exercising is borderline significant for the association between LDL-C variability and all-cause mortality.

We conducted several sensitivity analyses. The results were consistent when analysis was performed on multiply imputed data which properly handled missing lipid variability (*n* = 15,086), with generally attenuated HRs for all-cause and CVD mortality compared with the complete data method (*n* = 10,583). In addition, similar results were obtained after excluding 222 enrollees who died within 1 year after 3-year lipid profiles had been collected. When lipid quartiles (Q1–Q4) were used instead of continuous variable, the HRs of all-cause and CVD mortality were consistent for the highest quartile (Q4) compared with the lowest quartile (Q1) of HDL-C and LDL-C variability, as shown in Additional file 1: Table S1.

## Discussion

In our single-center cohort of 10,583 type 2 DM patients, HDL-C, LDL-C, and TC variability defined by CV was associated with all-cause and CVD mortality, while only HDL-C variability was associated with non-CVD mortality. These associations remained significant after multivariate adjustment for baseline lipid levels and traditional risk factors. Higher HDL-C variability consistently increased all-cause mortality in subgroups, while the association of LDL-C variability with all-cause mortality generally attenuated compared with that of TC variability. Our study indicates that visit-to-visit variability in blood lipid profiles can be useful in identifying type 2 diabetes patients with high mortality risk.

Although the exact mechanisms remain uncertain, some explanations to our results can be suggested. Studies have shown on-treatment variability in plasma lipids is associated with atherosclerotic plaque progression [25, 26]. Fluctuations of lipid levels may be detrimental to endothelium. Variability of lipid efflux may impair the stability of plaque, which in turn increases risk of plaque rupture. The dosing and adherence of statins may directly affect lipid variability in patients on treatment, but not exclusively [27, 28]. Abrupt discontinuation or intermittent use owing to myalgia side effect may be other reasons related to on-treatment lipid variability. To some extent, lipid variability may reflect medication strength and selfcare quality, both of which are related to health outcomes. However, studies have also shown that variability in LDL-C is associated with adverse cardiovascular outcomes, even after adjustment for lipid-lowering agents use [20] or medication adherence [28]. Lipid variability may be attributed to body weight change. Studies have found that fluctuation in body weight is a poor prognostic factor related to morbidity and mortality in the Framingham Study cohort [29]. Variability of cardiovascular risk factors is related to poor control of disease status, either pharmacologically or non-pharmacologically, and may thus indicate more adverse drug effects or complications. Aside from cardiovascular risk, lipid levels may also reflect nutrition status and body composition. Low LDL-C levels occur frequently in frail older people. Malnutrition affects “cholesterol paradox,” which attenuates or even reverses the adverse effect of elevated LDL-C levels, but that of LDL-C variability remains regardless of aging or undernutrition. Higher LDL-C variability has been related to higher urine protein-to-creatinine ratio in chronic kidney disease patients [30] and increased risk of atrial fibrillation [31].

The research on the effect of lipid variability on mortality can be traced to the Framingham Study cohort, which consisted of 2,912 subjects and found that TC variability assessed by root mean square error was positively associated with all-cause mortality in men and cardiovascular mortality in both sexes over a 24-year period [19]. Until recently, a Korean nationwide cohort study of 3,656,648 subjects showed that TC variability measured using CV was linearly associated with all-cause mortality, myocardial infarction (MI), and stroke during a median follow-up of 8.3 years. The authors also conducted a sensitivity analysis and found similar results when replacing CV with other variability indices like SD or variability independent of the mean (VIM) [20]. Another Korean study conducted among 1,792 subjects who underwent percutaneous coronary intervention further found that variability in LDL-C, HDL-C, and non-HDL-C was an independent predictor for all-cause mortality, MI, and stroke in patients undergoing percutaneous coronary intervention regardless of variability indices [21]. A retrospective cohort study in Hong Kong included 125,047 type 2 DM patients and found variability in LDL-C, TC to HDL-C ratio, and TG was linearly related to the risk of all-cause, CVD, and non-CVD mortality during a median follow-up of 6.5 years. They further analyzed subgroups by age and found that the hazardous effect of lipid variability on CVD and mortality was stronger for mid-aged subjects than the elderly [23]. However, a Korean nationwide cohort study of 1,934,324 statin-naive young adults found that abnormal baseline lipid profiles were significantly associated with increased risk of CVD, particular MI, but higher variability in lipid profiles measured by VIM was not consistently associated with increased risk of MI or stroke [32]. Our study focused on type 2 DM patients, and the study sample consisted of men and women around 59 years of age on average, who were slightly younger than the enrollees of the Hong Kong study. Our main results were generally consistent but complementary to the previous study, in that variability in lipid profiles except TG measured by CV was positively associated with all-cause and CVD mortality. Subgroup analysis demonstrated greater effect of TC variability on all-cause mortality in the non-elderly, which could be explained by older patients having more comorbidities that may mask the effect of lipid variability [23]. The HRs of all-cause mortality for TC and LDL-C variability were higher in patients without history of dyslipidemia. Lipid variability may be affected by the initiation and adherence of lipid lowering agents, and previous studies showed that the association of TC and LDL-C variability with all-cause mortality attenuated in individuals taking statins [20, 23]. Diabetes patients with dyslipidemia were likely to take medicine to lower blood lipids. However, this information for medication was lacking in our data.

HDL-C is recognized as a strong cardiovascular predictor. But beyond this lipid hypothesis, our results showed higher HDL-C variability was associated with all-cause and non-CVD mortality. Further subgroup analysis found higher HDL-C variability consistently increased all-cause mortality. In contrast, a cohort study showed higher HDL-C levels were related to higher risk of cardiovascular events and all-cause mortality in type 2 diabetes patients attaining LDL-C goals [33]. Other studies also reported the relationship between HDL-C and all-cause mortality followed U-shaped or J-shaped curves across levels of age, sex, and estimated glomerular filtration rate, in that very low or high HDL-C was associated with increased mortality risk [34, 35]. Though the mechanism for this nonlinear relationship remains unclear, evidence suggests that perhaps the increased risk of death is not solely explained by cardiovascular events. Intra-individual variability of HDL-C implies swings between two extremes and hence, the unfavorable results from increased HDL-C variability may be partly explained by a shift to more extreme values.

The strength of our study was comprehensive investigation of variability in lipid profiles as predictors of CVD and non-CVD deaths separately. Our results were drawn from a large single-center cohort of DCMP that represents type 2 DM patients in the primary care settings. Some limitations were also observed. The information of lipid-lowering agents use was unclear. Although the association of statins use with TC and LDL-C levels was well established, studies showed inconsistent results of the association between statins and lipid variability [28, 30]. TC variability was associated with the risk of all-cause mortality, MI, and stroke even after adjusting for mean TC levels and the use of lipid-lowering agents; however, interaction of TC variability and lipid-lowering agents with these adverse outcomes was found in Korean general population [20]. Although we confirmed the link between lipid variability and mortality, lipid variability may merely be a predictor rather than a causal factor, until assessing its role as target for intervention. In the end, we used CV as a summary measure of variability, instead of a full trajectory over time. This way was commonly adopted by previous literature, but it may oversimplify lipid fluctuation as some information had been discarded.

Future studies evaluating lifestyle modification, lipid-lowering agents use, and medication adherence enhancement, which affect lipid variability, are suggested. Investigating interventions targeting on reducing variability of lipids is important to evaluate effects of these interventions on health outcomes beyond the benefits of lowering lipid levels. Further head-to-head studies are also invited to evaluate between statins, focusing not only on achieved value but also on variability of lipids, due to lack of comparisons among different treatments.

## Conclusion

Lipid variability was independently associated with all-cause and CVD mortality in type 2 DM patients. The effect of TC and LDL-C variability on all-cause mortality was greater among subgroups of nonsmoking, regular exercising, absence of dyslipidemia, and more severe DM status at baseline.

## Supplementary Information

**Additional file 1: Table S1.** Hazard ratios of mortality for blood lipid variability categorized by quartiles in patients with type 2 diabetes. **Fig S1.** Flowchart for recruitment procedures.

## Data Availability

The datasets generated and/or analyzed during the current study are not publicly available due to the policy declared by National Health Insurance in Taiwan but are available from the corresponding author on reasonable request.

## Abbreviations

CVD: Cardiovascular disease
DM: Diabetes mellitus
TC: Total cholesterol
LDL-C: Low-density lipoprotein cholesterol
HDL-C: High-density lipoprotein cholesterol
TG: Triglycerides
SD: Standard deviation
DCMP: Diabetes Care Management Program
CMUH: China Medical University Hospital
ICD-9-CM: International Classification of Disease, 9th Revision, Clinical Modification
BMI: Body mass index
FPG: Fasting plasma glucose
HbA1c: Hemoglobin A1c
CV: Coefficient of variation
HRs: Hazard ratios
CIs: Confidence intervals
MI: Myocardial infarction
VIM: Variability independent of the mean
