# Supplementary material for: Effect of blood lipid variability on mortality in patients with type 2 diabetes: a large single-center cohort study
Source: Cardiovasc Diabetol. 2021 Nov 25;20:228. doi: 10.1186/s12933-021-01421-4 (PMC8620132; doi:10.1186/s12933-021-01421-4)
Supplement: Supplementary file 1 — Additional file 1: Table S1. Hazard ratios of mortality for blood lipid variability categorized by quartiles in patients with type 2 diabetes. Fig S1. Flowchart for recruitment procedures. [file 12933_2021_1421_MOESM1_ESM.docx]

**Additional file 1: Table S1.** Hazard ratios of mortality for blood lipid variability categorized by quartiles in patients with type 2 diabetes

|  | Q2 vs. Q1† | | Q3 vs. Q1† | | Q4 vs. Q1† | | |
| --- | --- | --- | --- | --- | --- | --- | --- |
|  | HR (95% CI)‡ | *p* value | HR (95% CI)‡ | *p* value | HR (95% CI)‡ | *p* value |  |
| All-cause mortality |  |  |  |  |  |  |  |
| HDL-C variability* | 0.88 (0.77, 1.02) | 0.08 | 1.03 (0.90, 1.18) | 0.66 | 1.36 (1.20, 1.54) | <0.001 |  |
| LDL-C variability* | 0.98 (0.87, 1.11) | 0.76 | 1.01 (0.89, 1.15) | 0.89 | 1.14 (1.00, 1.30) | 0.05 |  |
| TG variability* | 0.83 (0.73, 0.94) | 0.004 | 0.92 (0.81, 1.04) | 0.19 | 0.98 (0.86, 1.12) | 0.75 |  |
| TC variability* | 0.99 (0.87, 1.13) | 0.89 | 1.06 (0.93, 1.20) | 0.39 | 1.13 (0.98, 1.29) | 0.09 |  |
| Expanded CVD mortality |  |  |  |  |  | | |
| HDL-C variability* | 0.94 (0.76, 1.17) | 0.60 | 1.23 (1.00, 1.51) | 0.05 | 1.44 (1.18, 1.75) | <0.001 |  |
| LDL-C variability* | 0.95 (0.78, 1.15) | 0.58 | 1.04 (0.85, 1.27) | 0.73 | 1.28 (1.05, 1.55) | 0.01 |  |
| TG variability* | 0.78 (0.64, 0.95) | 0.02 | 1.04 (0.86, 1.26) | 0.66 | 1.13 (0.93, 1.37) | 0.23 |  |
| TC variability* | 1.04 (0.85, 1.26) | 0.72 | 1.15 (0.94, 1.40) | 0.17 | 1.28 (1.05, 1.57) | 0.02 |  |
| Non-expanded CVD mortality |  |  |  |  |  | | |
| HDL-C variability* | 0.83 (0.69, 1.00) | 0.05 | 0.90 (0.75, 1.08) | 0.25 | 1.32 (1.12, 1.56) | <0.001 |  |
| LDL-C variability* | 1.01 (0.86, 1.19) | 0.89 | 0.99 (0.83, 1.18) | 0.90 | 1.04 (0.87, 1.25) | 0.63 |  |
| TG variability* | 0.86 (0.73, 1.02) | 0.08 | 0.83 (0.70, 0.98) | 0.03 | 0.88 (0.74, 1.05) | 0.15 |  |
| TC variability* | 0.96 (0.81, 1.14) | 0.64 | 1.00 (0.84, 1.19) | 0.99 | 1.02 (0.85, 1.22) | 0.85 |  |

*Variability measured by coefficient of variation.

†The first quartile (Q1) serves as the reference for the other quartiles (Q2-Q4).

‡HRs adjusted for age, sex, smoking, alcohol drinking, exercising, BMI, duration of diabetes, types of diabetes treatment, FPG, HbA1c, HDL-C, LDL-C, TG, TC, hypertension, dyslipidemia, stroke, coronary artery disease, severe hypoglycemia, peripheral neuropathy, nephropathy, diabetic ketoacidosis, and hyperglycemic hyperosmolar nonketotic coma.

18,373 adults enrolled in the Diabetes Care Management Program

10,583 participants were included for analysis

17,421 subjects were eligible

Excluding

Type 1 diabetes or gestational diabetes (*n*=448)

Age<30 years old (*n*=504)

Enrollment period less than three year (*n*=6,385)

Without socio-demographic factors, life style behaviors, diabetes related factor, complications and blood biochemical indices (*n*=453)

**Additional file 1: Fig S1.** Flowchart for recruitment procedures.
